# Thermal Analysis of Copper-Titanium-Multiwall Carbon Nanotube Composites

**DOI:** 10.1186/s11671-017-2025-9

**Published:** 2017-04-04

**Authors:** Smail Hamamda, Ahmed Jari, S. Revo, K. Ivanenko, Youcef Jari, T. Avramenko

**Affiliations:** 1 Laboratory of Thermodynamics and Surface Treatment of Materials, University of Frères Mentouri Constantine 1, B.P. 325 Route, Ain El Bey, Constantine 25017 Algeria; 2 R&D Laboratory of Metal and Ceramics Physics, Taras Shevchenko National University of Kyiv, 64/13, Volodymyrska Street, 01601 Kyiv, Ukraine

**Keywords:** Copper, Titanium, Nanotube, Spectroscopy, Thermal expansion

## Abstract

The aim of this research is the thermostructural study of Cu-Ti, Cu-Ti 1 vol% multiwall carbon nanotubes (MWCNTs) and Cu-Ti 3 vol% MWCNTs. Several investigation techniques were used to achieve this objective. Dilatometric data show that the coefficient of thermal expansion of the nanocomposite containing less multiwall carbon nanotubes is linear and small. The same nanocomposite exhibits regular heat transfer and weak mass exchange with the environment. Raman spectroscopy shows that the nanocomposite with more MWCNTs contains more defects. This implies that the carbon nanotubes have better dispersion in Cu-Ti 1 vol% MWCNTs. Infrared spectroscopy reveals that Cu-Ti 1 vol% MWCNTs has better crystallinity than Cu-Ti 3 vol% MWCNTs.

## Background

Modern life and industry increasing needs require materials and investigation techniques that offer better performance and cost-effectiveness. The emerging technologies compel scientists to relentlessly seek innovation in designing new processes and new techniques that are more efficient, more practical, offering longer lifetime or suitable for use in adverse conditions, with higher constraints or in corrosive settings where temperature gradients are extreme. Nowadays, climatic disturbances which may repeatedly result in natural disasters must be taken into account more seriously.

Advances in mastery of new techniques, allowing the design of new products showing exceptional physical properties, made it possible to substitute heavy alloys, expensive and environmentally unfriendly, with nanocomposites containing carbon nanotubes (CNTs). The published literature about nanomaterials is unanimous in hailing the performance and the positive role of CNTs in polymer or metallic matrices [1, 2].

Since their discovery, CNTs became the first choice in many applications [3, 4]. They exhibit great potential, and their possible uses are constantly expanding due to their very large aspect ratio, high rigidity, high resistance to traction, virtual absence of thermal expansion, excellent electrical conductivity and good thermal conductivity. These characteristics make them compellingly useful in the preparation of nanocomposites [5].

Many articles have been published about aluminium-based nanocomposites. Their authors unanimously point out an improvement of the mechanical, thermal and electrical properties and a significant decrease in the coefficient of thermal expansion of the material through the incorporation of CNTs. Similarly, the incorporation of CNTs into polymer matrices improves the percolation threshold and the electrical and tribological properties of the material. Recent studies in Refs. [1, 2] have demonstrated that the introduction of CNTs into polymer or metal matrices causes a decrease in thermal expansion and anisotropy.

Several studies have been devoted to copper-based composites for their interesting physical properties: a small coefficient of thermal expansion and high electrical and thermal conductivities [6]. They are competitively energy-wise, and their use in various applications is expanding. Strengthening these matrices with nanotubes has become very attractive to respond to different demands, e.g. producing cleaner energy, reducing pollution, improving the value-for-money ratio.

Interest in the class of copper-based nanocomposites is increasing [7]. Addition of CNTs results in excellent characteristics, notably, high electrical conductivity and Young modulus [8]. CNTs should allow the improvement of mechanical and electrical properties through their homogeny distribution in the matrix.

Research has shown that heat flow in Cu-CNTs is limited by the weak bonds across the Cu-CNT interfaces and that its thermal conductivity is significantly lower than the estimated value for the intrinsic thermal conductivity of the CNTs.

These shortcomings are probably due to the discrepancy between the coefficient of thermal expansion of the CNTs and that of copper and the weak bond between the NTCs and Cu. Some authors have shown that the insertion of carbide-forming elements, such as Ti, Cr or Zr, improves the mechanical and thermal characteristics of Cu-CNT nanocomposites as well as the interfacial bond between Cu and the CNTs [9].

In this study, titanium was inserted into a Cu-CNT nanocomposite. Titanium has been chosen because microstructural analysis has shown that the formation of TiC layers considerably improves the interfacial bond between the CNTs and the copper matrix [9]. The thermal conductivity of Cu-Ti-CNTs is significantly higher than that of Cu-CNTs [10]. The authors [11, 12] confirmed the positive role of carbides.

The published literature deals mainly with the electrical and mechanical properties of Cu-Ti-CNTs. It has shown that addition of Ti improves the electrical conductivity [10]. Authors of Ref. [10] have confirmed the increase in the elastic limit, improving the Cu limit by 115% and the Cu-CNT limit by 88%. In Ref. [13], the authors observed that the mechanical properties of Cu-Ti-CNT nanocomposites improved. Microhardness tripled and the elastic limit doubled their values compared to copper. This improvement has attributed to the use of a very-high-energy planetary ball mill, which induced a change in the grain size that resulted in a higher density of grain boundaries and thus a more effective block against the movement of dislocations.

This study aims at contributing in closing the gap in research on the thermal expansion and structural characteristics of Cu-Ti-CNT nanocomposites, as well as the influence of the CNT concentration on these characteristics.

This research is a thermostructural study of the Cu-Ti-CNT nanocomposites with different concentrations of multiwall CNTs. The objective is to analyse the correlation between the structural and thermodynamic properties.

## Methods

The nanocomposites were obtained using PMS-1 copper [14] and VT1-0 titanium [15] powders. Multiwall carbon nanotubes [16] (MWCNTs) were prepared in a rotating disk CVD reactor [17]. The catalyst used to produce the MWCNTs was an oxide mixture containing Al_2_O_3_, MoO_3_ and Fe_2_O_3_. The carbon source was propylene. The average diameter of the CNTs is comprised between 10 and 20 nm. The specific area, determined through Ar desorption, is of the order of 200 to 400 m^2^/g. The apparent volumetric mass density is 20 to 40 g/dm^3^.

The nanocomposites were elaborated in a planetary ball mill with an acceleration of 50 g, for 20 min, then annealed three times at 950 °C for 30 min, pressed three times by 40% and rolled one time by 80% [13].

Three materials are the object of this study. They all contain the same concentration of titanium (1 wt%). The first is a pure copper matrix with titanium additions (1 wt%). The second, Cu-Ti 1 vol% MWCNTs, is Cu 1 wt% Ti to which 1 vol% of multiwall carbon nanotubes were added. The third material, Cu-Ti 3 vol% MWCNTs, has a higher concentration of MWCNTs (3 vol%) [13, 18].

The following characterization techniques have been used:

- Thermal expansion was measured with a Netsch 402C dilatometer. *α*(*T*) was measured along the direction perpendicular to the rolling plane of each film. The heating rate is invariably 10 °C/min. The temperature range considered is 25–750 °C.
- Differential scanning calorimetry (DSC) and thermogravimetric (TG) tests were conducted in a Jupiter STA 449F3 calorimeter manufactured by Netsch. The heating rate is identical with that used for dilatometric tests, namely, 10 °C/min.
- Spectra were obtained with a Jasco FT/IR-6300, for infrared spectroscopy, and a Bruker Senterra, for Raman spectroscopy.

## Results and Discussion

**Fig. 1:**
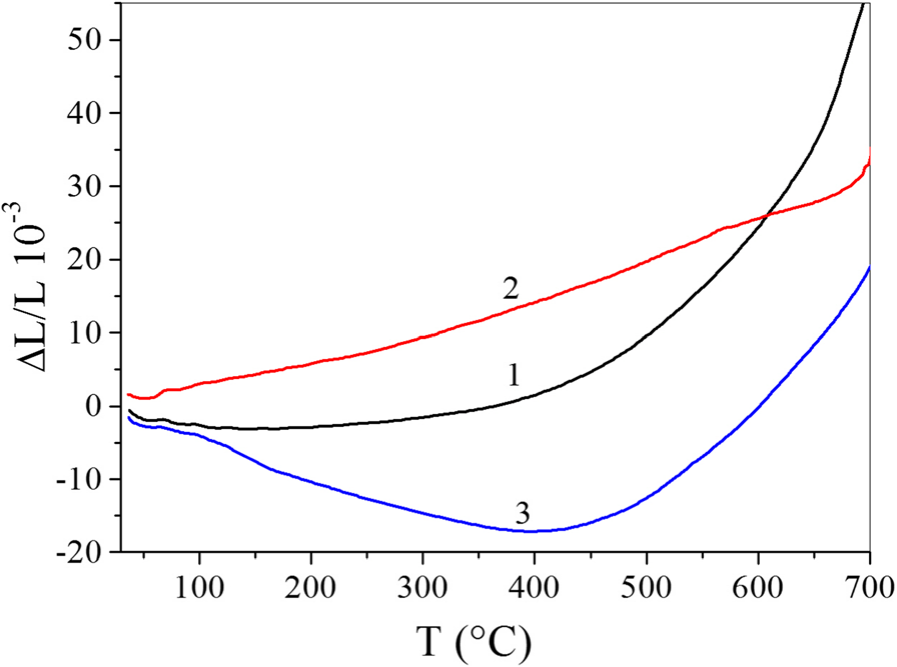
The relative variation in length, Δ*L*/*L*, with temperature of the three materials: Cu-Ti (*1*), Cu-Ti 1 vol% MWCNTs (*2*) and Cu-Ti 3 vol% MWCNTs (*3*)

The relative variation in length, Δ*L*/*L*, in the three materials are clearly distinct (Fig. 1).

The MWCNTs do not have the same effect on the expansion behaviour of the samples. The amount of MWCNTs in each nanocomposite reveals its specificity. The shapes of Δ*L*/*L* for Cu-Ti and Cu-Ti 3 vol% MWCNTs are similar. The two curves exhibit an extended minimum around 400 °C. This peak is 20 times more intense in Cu-Ti 3 vol% MWCNTs than it is in Cu-Ti.

The Δ*L*/*L* of Cu-Ti 3 vol% MWCNTs is lower than those of the two other materials over the whole temperature range. At 500 °C, Δ*L*_1_/*L* is six times higher than Δ*L*_3_/*L* while Δ*L*_0_/*L* is only four times higher (the superscripts indicate the concentration of MWCNTs, in vol%). Around 600 °C, Δ*L*_1_/*L* and Δ*L*_0_/*L* intersect. We observe that Δ*L*_0_/*L* is very intense while Δ*L*_1_/*L* varies monotonously over the whole temperature interval with a very weak slope compared to those of the two other materials.

**Fig. 2:**
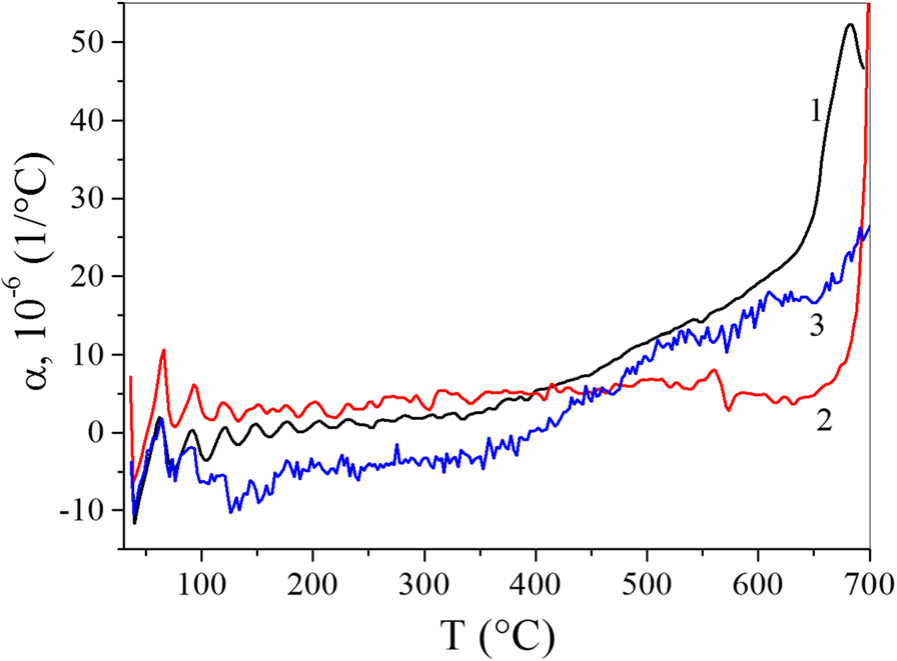
Variation with temperature of the coefficient of thermal expansion measured in the direction perpendicular to the rolling plane of Cu-Ti (*1*), Cu-Ti 1 vol% MWCNTs (*2*) and Cu-Ti 3 vol% MWCNTs (*3*)

Figure 2 shows the variation with temperature of the coefficient of thermal expansion measured in the direction perpendicular to the deposition plane for the three materials.

The shapes of the three curves are similar. The variation of *α*(*T*) can be divided into three distinct regions. From room temperature to 400 °C, *α*_0_(*T*) is between *α*_1_(*T*) and *α*_3_(*T*) and *α*_3_(*T*) is smaller than the two other coefficients. Starting from 400 °C, the trend is reversed. *α*_0_(*T*) increases considerably and becomes larger than both *α*_1_(*T*) and *α*_3_(*T*). *α*_3_(*T*) increases a little, but *α*_1_(*T*) remains virtually unchanged. Beyond 650 °C, the intensities of the *α*_*i*_(*T*) become very significant. Oxidation probably affects the samples. At lower temperatures, *α*_3_(*T*) is slightly negative. This behaviour may be related to the significant concentration of MWCNTs in the material.

We observe that the incorporation of 1% of CNTs into the Cu-Ti matrix results in a nanocomposite with a constant expansion over a large (~500 °C) temperature interval. The mean value of the coefficient of thermal expansion over this temperature interval is 4 × 10^−6^ °C^−1^. This value is of the same order of magnitude as the *α*(*T*) of the CNTs [19]. This stability in the thermal expansion means that the interactions in the nanocomposite are virtually the same.

**Fig. 3:**
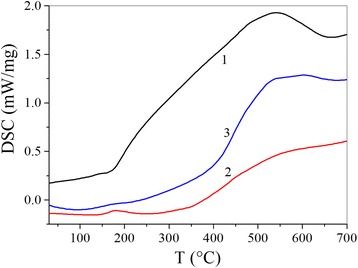
Differential scanning calorimetry curves of Cu-Ti (*1*), Cu-Ti 1 vol% MWCNTs (*2*) and Cu-Ti 3 vol% MWCNTs (*3*)

Figure 3 shows the DSC curves for the three materials.

The shape of the calorimetric curves depends on the concentration of MWCNTs. The calorimetric behaviours of Cu-Ti 3 vol% MWCNTs and Cu-Ti are similar. Both curves show a singularity around 570 °C with a different intensity in each curve. The ratio of the two intensities is 0.8. As to Cu-Ti 1 vol% MWCNTs, its calorimetric behaviour is completely different from the other two materials. There is no singularity visible on the curve. From room temperature to 330 °C, the curve is linear. Over the rest of the interval, it increases slightly. The MWCNTs are the source of a radical change in the calorimetric behaviour of the material. When the amount incorporated is high (3 vol% CNTs), the DSC signal intensifies. The release of heat is significant. However, a smaller addition (1 vol% CNTs) does not affect the signal in any significant way. The exothermic effect is very small. It is even less intense than in the material with no nanotubes. At high temperatures, the DSC signal is virtually constant.

**Fig. 4:**
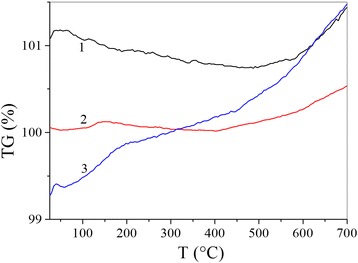
Thermogravimetric curves of Cu-Ti (*1*), Cu-Ti 1 vol% MWCNTs (*2*) and Cu-Ti 3 vol.% MWCNTs (*3*)

In a similar fashion, the shape of the thermogravimetric curves depends on the concentration of nanotubes (Fig. 4).

The TG curve for Cu-Ti exhibits a sharp slope. At high temperatures, the relative mass variation for Cu-Ti and Cu-Ti 3 vol% MWCNTs increases considerably. Starting from 600 °C, they are superimposed and then completely “fuse” together. This is due to the fact that oxidation has the same effect on the two materials. However, the TG curve for Cu-Ti 1 vol% MWCNTs is essentially lower over a large interval of temperatures; no net mass transfer occurs in the sample. Starting from 500 °C, we observe that the change in slope on the TG curve is small if compared to the curves of the two other materials.

**Fig. 5:**
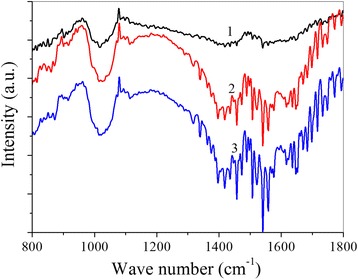
Infrared spectra of Cu-Ti (*1*), Cu-Ti 1 vol% MWCNTs (*2*) and Cu-Ti 3 vol% MWCNTs (*3*)

Infrared spectroscopy shows that for compositions with different concentrations of MWCNTs, spectra have differences (Fig. 5).

For example, the spectrum of the material with higher nanotube content shows very wide bands and intense peaks [20–23]. However, for Cu-Ti 1 vol% MWCNTs, the peaks are less intense and the bands tend to be narrower while they appear at the same frequencies as in Cu-Ti 3 vol% MWCNTs. This indicates that the incorporation of 1% MWCNTs improves the crystallinity of the material. The Cu-Ti spectrum exhibits very weak singularities at frequencies that coincide with those in the two nanocomposites.

## Conclusions

The results obtained in this research confirm the positive role of carbon nanotubes in materials. The thermostructural properties of the nanocomposites studied depend on the concentration of carbon nanotubes.

The incorporation of 1 vol% of MWCNTs in a Cu-Ti matrix results in more interesting thermodynamic and structural properties when compared with those of the material containing 3 vol% MWCNTs. Cu-Ti 1 vol% MWCNTs has a coefficient of thermal expansion that is linear and small, a nearly constant heat capacity and negligible mass loss over a large temperature interval.

Infrared spectroscopy shows that Cu-Ti 1 vol% MWCNTs has better crystallinity than Cu-Ti 3 vol% MWCNTs.

The range of intensities changes for Δ*L*/*L* = *f*(*T*), and *α* = *f*(*T*) parameters (Figs. 1 and 2) can be divided into three parts: the area *T* < 400 °C, where Δ*L*/*L* and *α* change slightly and areas *T* = 400–600 and 600–700 °C, with significant changes of mentioned parameters. A characteristic feature is that the value of Δ*L*/*L* is substantially less for Cu-Ti and Cu-Ti—1% CNT compositions than for the Cu-Ti—3% CNT composition in the entire temperature range.

The addition of carbon nanotubes to the Cu-Ti composition up to 3 vol% reduces the magnitude of *α*(*T*). For example, in the temperature range up to *T* = 400 °C, it is lower for both the Cu-Ti composition without nanotubes and the composition with 1 vol% of carbon nanotubes. At *T* = 400–700 °C, the increasing of *α*(*T*) for all tested samples was observed. These changes were also lower for compositions with nanotubes than for the samples without them. Effect of nanotubes in this case is caused that their expansion coefficient is close to zero.

Addition of nanotubes in the studied composition radically changes its calorimetric and thermogravimetric behaviour. Changing the concentration of nanotubes in the investigated compositions and measurement results of their behaviour allows obtaining materials with predetermined characteristics. Aside from these features for the compositions with nanotubes, infrared and Raman spectra evolve.

## Abbreviations

CNTs: Carbon nanotubes
CVD: Chemical vapour deposition
DSC: Differential scanning calorimetry
MWCNTs: Multiwall carbon nanotubes
TG: Thermogravimetric
